# LiNi_0.8_Co_0.15_Al_0.05_O_2_: Enhanced Electrochemical Performance From Reduced Cationic Disordering in Li Slab

**DOI:** 10.1038/s41598-017-01657-9

**Published:** 2017-05-03

**Authors:** Peng Xiao, Tingjian Lv, Xueping Chen, Chengkang Chang

**Affiliations:** School of Materials Science and Engineering, Shanghai Institute of Technology, 100 Haiquan Road, Shanghai, 201418 China

## Abstract

Sub-micron sized LiNi_0.8_Co_0.15_Al_0.05_O_2_ cathode materials with improved electrochemical performance caused by the reduced cationic disordering in Li slab were synthesized through a solid state reaction routine. In a typical process, spherical precursor powder was prepared by spray drying of a uniform suspension obtained from the ball-milling of the mixture of the starting raw materials. Then the precursor powders were pressed into tablets under different pressures and crushed into powder. The pressing treated powders were finally calcinated under oxygen atmosphere to obtain the target cathode materials. XRD investigation revealed a hexagonal layered structure without impurity phase for all samples and significant increase in the diffraction intensity ratio of I_(003)_/I_(104)_ was observed. Rietveld refinement further confirmed the reduced cationic disordering in Li slab by such pressing treatment, and the smallest disordering was observed for sample S4 with only 1.3% Ni ions on Li lattice position. The electrochemical testing showed an improvement in electrochemical behavior for those pressing treated samples. The calculation of diffusion coefficients using EIS data showed improved Li diffusion coefficient after pressing treatment. The sample S4 presented a diffusion coefficient of 4.36 × 10^−11^ cm^2^·s^−1^, which is almost 3.5 times the value of untreated sample.

## Introduction

Layered LiNi_0.8_Co_0.15_Al_0.05_O_2_ cathode materials is considered the most promising of the next generation cathode materials for HEV and PHEV due to its high energy density and low toxicity^1–3^. However, some of drawbacks seem to restrict its commercialization, which includes: (1) the traditional synthesis method needs further development. The commercial way to prepare LiNi_0.8_Co_0.15_Al_0.05_O_2_ is the precursor method in which co-precipitated salts mixed with lithium source was calcinated^4, 5^. This method has two shortcomings. Firstly, it is hard to obtain a uniform distribution of elements of the precursor because there is a huge gap between the Ksp of Ni(OH)_2_, Co(OH)_2_ and Al(OH)_3_ (2.0 × 10^−15^, 5.92 × 10^−15^and 1.3 × 10^−33^ respectively). Secondly, this process will produce a large number of acid ions containing waste water, resulting in environmental pollution and increasing production costs. (2) Cationic mixing in the Li slab limits the electrochemical performance of the NCA cathode material. In the commercial synthesis process, since it is very difficult to oxidize Ni^2+^ into Ni^3+^, cationic disordering is observed for such layered cathode materials, leading to the formation of a lithium deficient cathode material $({{\rm{Li}}}_{{\rm{1}}-{\rm{x}}}{{\rm{Ni}}}_{{\rm{x}}}^{{\rm{2}}+})({{\rm{Ni}}}_{{\rm{x}}}^{{\rm{2}}+}{{\rm{Ni}}}_{{\rm{1}}-{\rm{2x}}}^{{\rm{3}}+}){{\rm{O}}}_{2}$ ^6–8^. The Ni^2+^ in the Li site will be oxidized to Ni^3+^ during the electrochemical charging, which leads to the collapse of layered structure around Ni^3+^ ions since the radius of Ni^3+^ is smaller than that of Li^+^, thus further results in a huge polarization loss in the specific capacity.

It is reported that the use of surface modification methods (LiCo_2_^9, 10^, Al_2_O_3_^11^, LiMnPO_4_^3^, ZnO^12^, FeF_3_^13^, TiO_2_^14^,) can effectively solve the above-mentioned problems. The coating layer prevents the contact of the active material with the electrolyte. The Mg^2+^and F^−^ doping can restrain the cationic mixing in a certain extent^15–18^. However, there are few ways to solve above problems at the same time by optimizing the synthesis method. Xie, H^19^ *et al*. reported a synthesis method with 5-sulfosalicylic acid as a chelating agent to solve the ion pollution. But there are still some inherent shortcomings of such improved co-precipitation method, such as the complex synthesis process and the large amounts of waste water produced by the rinsing procedure.

Herein, we introduce a new method for synthesis of submicron sized LiNi_0.8_Co_0.15_Al_0.05_O_2_ with effectively reduced cationic mixing together with the electrochemical behavior investigation of the prepared powder. The powder was obtained by calcinating a precursor powder with pressing treatment under different pressure, and the electrochemical tests indicated enhanced specific capacity and capacity retention due to the improved Li ion diffusion caused by the reduced cationic disordering. The submicron sized NCA materials obtained by the pressing treatment routine illustrated very attractive electrochemical performance for future application.

## Methods

### Synthesis of the cathode materials

LiNi_0.8_Co_0.15_Al_0.05_O_2_ cathode materials were synthesized through a solid state reaction routine in which a precursor powder was calcined under flowing O_2_ atmosphere. The precursor powder is prepared via a ball milling-spray drying process. In a typical process, raw materials employed in the experiment are Nickel oxide (99%), Cobalt oxide (99.9%), Aluminum oxide (99.9%) and Lithium carbonate (99%), all in analytical grade. All the starting raw materials, with certain molar ratio (Li:Ni:Co:Al = 1.05;0.85:0.15:0.05), were mixed with deionizer water, then milled for 4hrs. The obtained slurry was dried by a spray dryer to form spherical precursors with particle size between 2–5 μm. Then the precursor powders were pressed into tablets under 2 MPa, 4 MPa and 6 MPa, followed by crushing and sieving (200 meshes) in room temperature. Finally, the powders were calcined under 750 °C for 12 h in oxygen atmosphere to obtain the target LiNi_0.8_Co_0.15_Al_0.05_O_2_ cathode material. The samples prepared without pressing treatment is denoted as sample S0, while the other samples press treated are denoted as S2, S4 and S6 for 2 MPa, 4 MPa, 6 MPa treated sample respectively.

### Characterization of the prepared materials

The crystal structures of samples were determined by TD3200 X-ray diffraction (40KV, 30mA, Cu Kα1 radiation). The data were collected in the 10°–70° (2θ) range using 0.01° (2θ) steps to the structural study with Rietveld refinement by Jade6.5. The chemical composition of samples was evaluated by inductively coupled plasma atomic emission spectroscopy test (ICP-AES). The morphologies of prepared samples were observed via a scanning electron microscopy (SEM, Hitachi, SU8200).

### Electrochemical testing

The electrochemical performances of the samples were measured using electrodes CR2016 coin-type half cells, which were assembled inside a glove box filled with Ar. A mixture of the calcined powders, Super P carbon black and PVDF (in the weight ratio of 8:1:1), dispersed by NMP, was pasted on aluminum foil, then dried in vacuum at 110 °C for 12 h. The cathode electrodes were pinched from the film into a disc with diameter of 12 mm and the average loading density of active materials for every positive electrode was 9.76 mg·cm^−2^. Then assemble with Li foils using Celgard 3501 as the separator. The cells were charged and discharged on a Land CT2001 battery tester at the voltage of 2.8–4.3 V. Electrochemical Impedance Spectroscopy (EIS) was conducted by an electrochemical workstation (Autolab Pgstat302n) over a frequency range of 0.05–500 KHz. Cycle votammetry (CV) of the cells were measured by CHI660B electrochemical measurement system.

## Result and Discussion

### Physical characteristics

**Figure 1:**
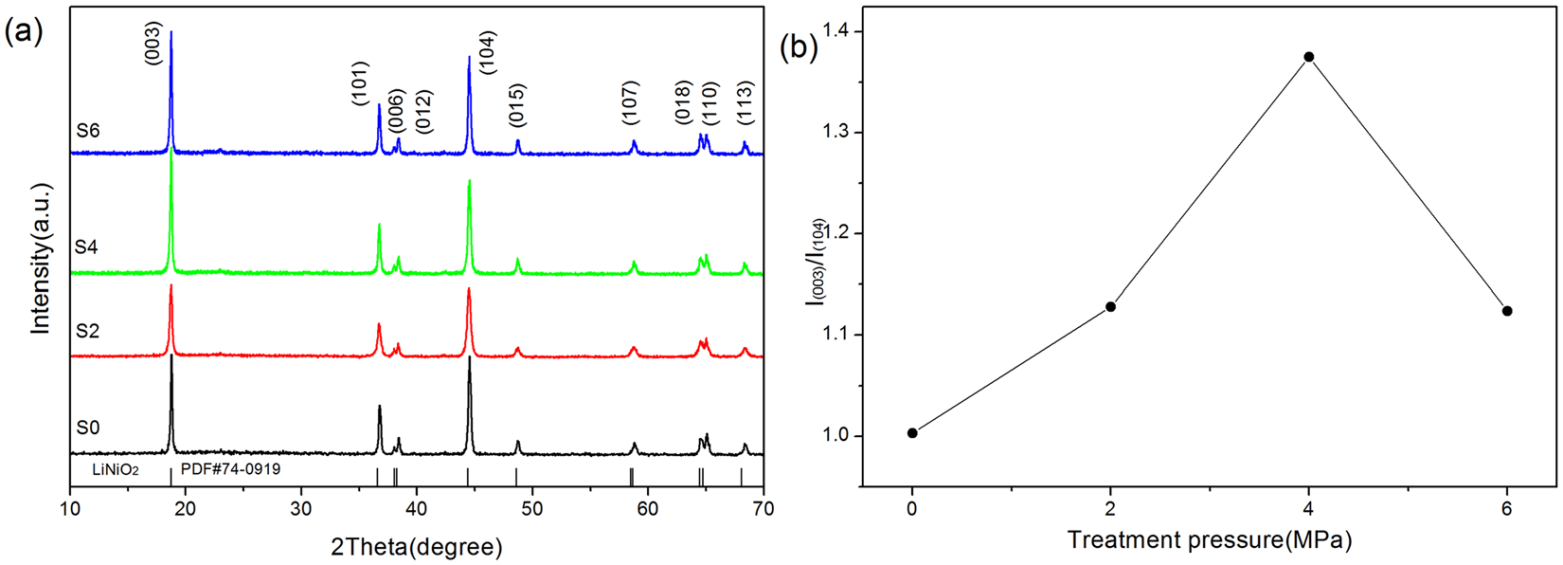
(**a**) XRD patterns of all the samples, (**b**) the relationship between the pressure and the value of I_(003)_/I_(104)_.

The XRD patterns of the prepared samples are show in Fig. 1 (a). All the diffraction peaks of samples matched well with that of standard pattern of LiNiO_2_ (PDF#74-0919) and therefore can be indexed into the layered structure of α-NaFeO_2_ (R-3m space group)^20^ without other impure phase. It can be observed clearly from the patterns the splitting of the (006)/(102) and (018)/(110) doublets at 40° and 65°, which indicated a highly ordered layered structure for the prepared cathode materials. Furthermore, a significant change in diffraction intensity ratio of I_(003)_/I_(104)_ is observed. It has been regarded that the intensity ratio of I_(003)_/I_(104)_ is an important index for the evaluation of the electrochemical performance of such layered cathodes, and high ratio value usually means good electrochemical performance. Figure 1(b) further compared the intensity ratios for sample S0–S6. It can be seen from Fig. 1(b) that the intensity ratio of I_(003)_/I_(104)_ is 1.003, 1.128, 1.375 and 1.124 with the pressure of treatment increasing from 0MPa to 6MPa, among which the sample S4 presented the highest intensity ratio. It was reported by the other groups that the intensity ratio can be regarded as an indicator of the ion mixing within the Li Slab, and a higher ratio of I_(003)_/I_(104)_ represents a better degree of ordering in Li slab^6, 21^. Majumder, S. B^7^ and Li, X^18^ reported high performance NCA materials with intensity ratio of 1.28 and 1.12 respectively. In our approach, we prepared NCA cathode powder with maximum intensity ratio of 1.375 through such a simple routine, which is more advantageous compared with previous works and therefore good electrochemical performance is expectable.

**Figure 2:**
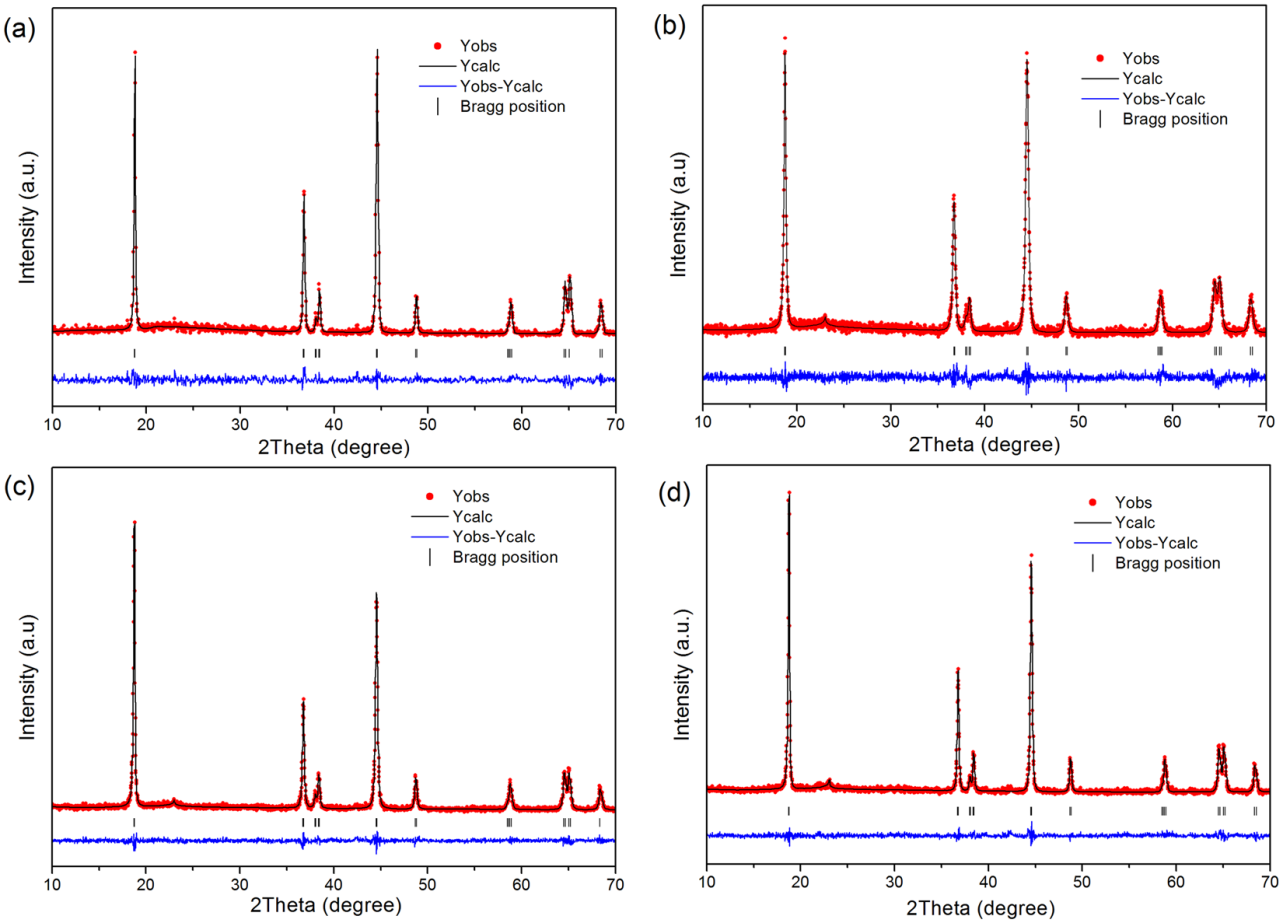
Rietveld refinement of samples: (**a**) S0, (**b**) S2, (**c**) S4 and (**d**) S6.

To demonstrate such pressing- treatment helps to reduce the degree of Li and Ni mixing, Rietveld refinements were conducted for the four powder sample with Jade6.5 software, and the results are showed in Fig. 2 and listed in Table 1. The weight profiled factor Rwp and confidence factor R in Table 1 are two important factors to evaluate the refinement results, and it is reliable and acceptable when the R factor are below 10%. Therefore, in our case, the small weight profiled factor Rwp and confidence factor R demonstrated the proposed structural model is correct, and the refinement results are acceptable. As seen from the Table 1, the lattice parameters for the four samples varied very slight, but the values of Ni in Li lattice position changed significantly. It can be seen from the table that, with increasing pressure, the disordering of Ni in Li site changes. The values are 5.4%, 3.7%, 1.3% and 3.4%, respectively. The amounts of disordered nickel in the lithium position of S4 sample reduced by 4.1% when compared to that of sample S0, further demonstrated that a low degree of cationic mixing is achieved by such a simple pressing treatment.

**Table 1:** Result of structural Analysis Obtained from X-ray Rietveld refinement of the materials.

S0	a(Å)	2.8632	c(Å)	14.1776	V(Å^3^)	100.7	Error Analysis	
	Li	Ni1	Ni2	Al	Co	O	Rwp(%)	R(%)
Site	3b	3b	3a	3a	3a	6c	9.52	7.02
Position	(0, 0, 1/2)	(0, 0, 1/2)	(0, 0, 0)	(0, 0, 0)	(0, 0, 0)	(0, 0, 0.2557)		
Occupancy	0.956	0.054	0.750	0.056	0.153	0.988		
**S2**	**a(Å)**	**2.8647**	**c(Å)**	**14.1982**	**V(Å** ^**3**^ **)**	**101.1**	**Error Analysis**	
	**Li**	**Ni1**	**Ni2**	**Al**	**Co**	**O**	**Rwp(%)**	**R(%)**
Site	3b	3b	3a	3a	3a	6c	9.29	7.36
Position	(0, 0, 1/2)	(0, 0, 1/2)	(0, 0, 0)	(0, 0, 0)	(0, 0, 0)	(0, 0, 0.2555)		
Occupancy	0.963	0.037	0.766	0.052	0.152	0.979		
**S4**	**a(Å)**	**2.8651**	**c(Å)**	**14.1883**	**V(Å** ^**3**^ **)**	**100.9**	**Error Analysis**	
	**Li**	**Ni1**	**Ni2**	**Al**	**Co**	**O**	**Rwp(%)**	**R(%)**
Site	3b	3b	3a	3a	3a	6c	8.69	7.82
Position	(0, 0, 1/2)	(0, 0, 1/2)	(0, 0, 0)	(0, 0, 0)	(0, 0, 0)	(0, 0, 0.2554)		
Occupancy	0.987	0.013	0.792	0.057	0.152	0.999		
**S6**	**a(Å)**	**2.8652**	**c(Å)**	**14.1918**	**V(Å** ^**3**^ **)**	**100.9**	**Error Analysis**	
	**Li**	**Ni1**	**Ni2**	**Al**	**Co**	**O**	**Rwp(%)**	**R(%)**
Site	3b	3b	3a	3a	3a	6c	8.48	7.64
Position	(0, 0, 1/2)	(0, 0, 1/2)	(0, 0, 0)	(0, 0, 0)	(0, 0, 0)	(0, 0, 0.2557)		
Occupancy	0.966	0.034	0.768	0.052	0.150	0.978		

Chemical compositions of all samples are also determined by ICP-AES, as shown in Table 2. The result shows the ICP elemental results of all samples are very close to the nominal composition and the results from the XRD refinements. Which demonstrate press treatment does not change obviously the proportion of each element in sample, and further verify the reliability of the refinement.

**Table 2:** chemical compositions for all samples.

	Mol ratio	Li	Ni	Co	Al
S0	nominal composition	1.050	0.800	0.150	0.050
	ICP result	0.955	0.802	0.151	0.048
	XRD refinement result	0.956	0.804	0.153	0.056
S2	nominal composition	1.050	0.800	0.150	0.050
	ICP result	0.967	0.804	0.158	0.049
	XRD refinement result	0.963	0.803	0.152	0.052
S4	nominal composition	1.050	0.800	0.150	0.050
	ICP result	0.985	0.802	0.156	0.048
	XRD refinement result	0.987	0.805	0.152	0.057
S6	nominal composition	1.050	0.800	0.150	0.050
	ICP result	0.968	0.804	0.153	0.049
	XRD refinement result	0.966	0.802	0.150	0.052

The precursor powder was prepared by sprays drying, through which hollow spherical particles were obtained. For such a hollow structure, the average distance between the raw material particles is considered relatively long. With pressure treatment, the average distance is reduced significantly. The final state of the NCA crystals for the different 4 samples (mainly the cationic misplacement) is determined by two major factors, the rate of source supplement and the rate of crystal growth. The source supplement rate is controlled by the diffusion process in which the cations migrate over the average distance to the surface of the NCA crystallites. The growth rate however is controlled by the adjusting of ionic occupancy to form the hexagonal structure. Therefore, the final structure of NCA crystals is determined by the competition results of the two processes. When the pressure is less than a certain value, the source supplement rate is not high enough and the growth process is dominant. Therefore, with increasing pressure, reduced disordering is achieved. However, at high pressure, the source supplement rate is high enough, and a large amount of cations is transported to the surface of the NCA crystallites which make it hard to form a “perfect” crystal in very short time, and nickel ions will enter the lithium slab filling the lithium vacancies. Cationic misplacement appears again at this circumstance.

**Figure 3:**
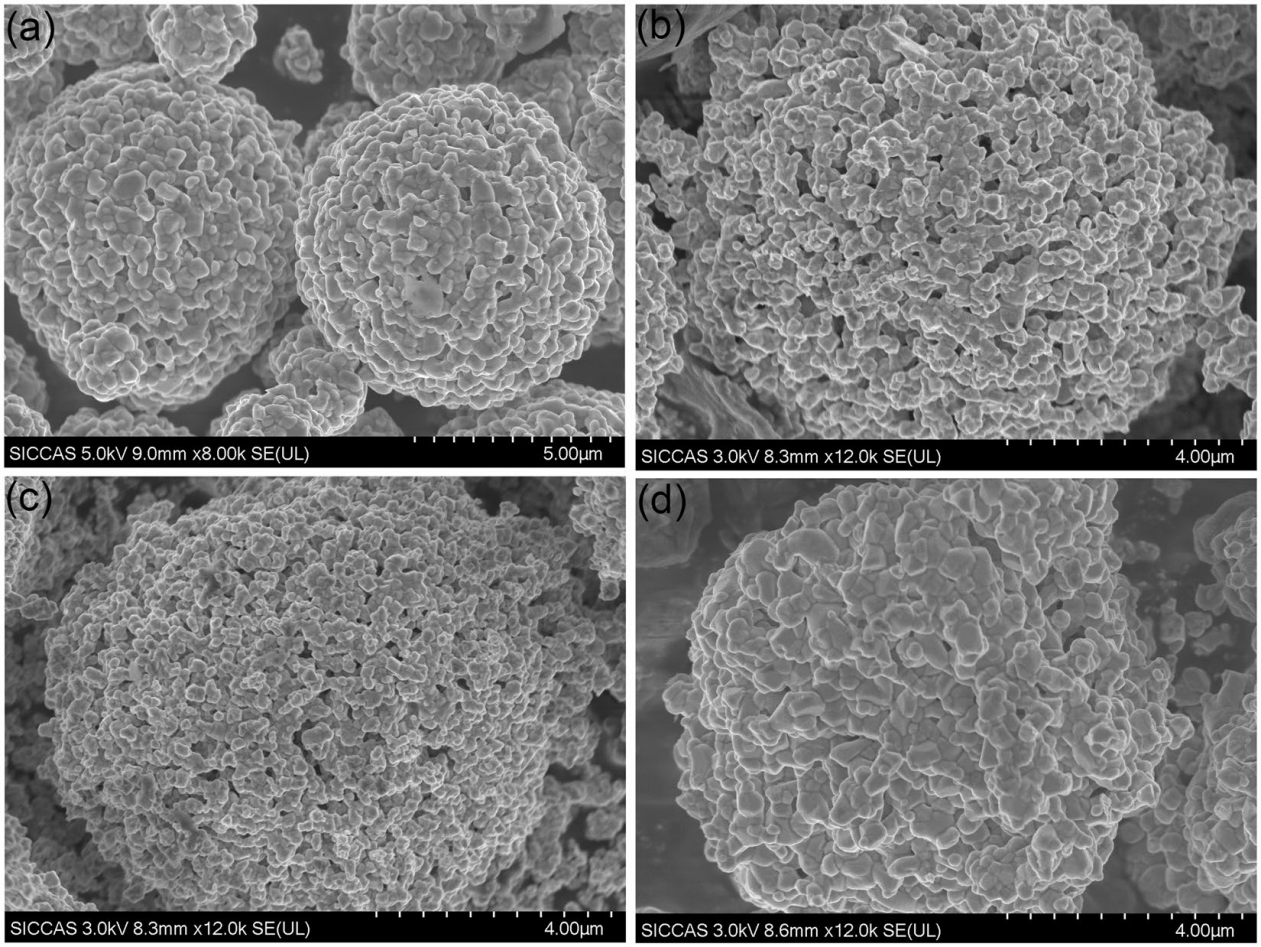
SEM images of cathode samples (**a**) S0, (**b**) S2, (**c**) S4 and (**d**) S6.

Figure 3 shows the SEM images of the different four NCA samples. All the samples are comprised by secondary particles with diameter about 4–6 μm, which were aggregated by tiny primary particles with size around 200–500 nm. It also can be seen from the micrographs that, with the increasing in treatment pressure, no obvious size change was observed for all the samples, which means that the pressing treatment will not significantly alter the size of the final NCA crystallites. This suggests that the observed difference in electrochemical performance is not caused by the difference in particle size.

### Electrochemical properties

**Figure 4:**
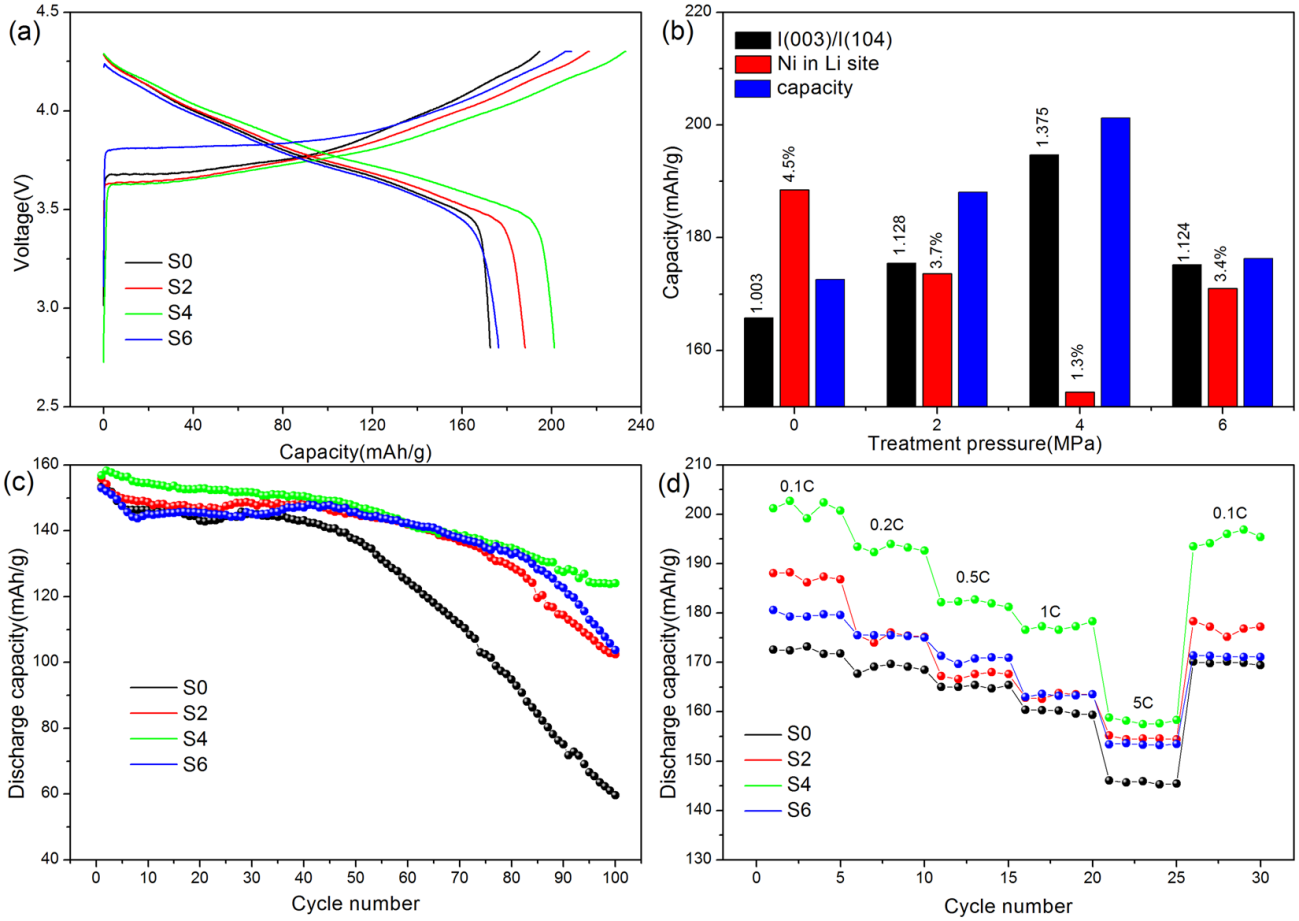
(**a**) Initial charge and discharge curves at 0.5C rate; (**b**) relationship among the I_(003)_/I_(104)_, cationic disordering and the discharge capacity; (**c**) cyclic performance at 5C rate; (**d**) rate performance of samples.

The differences in electrochemical performance for the samples were investigated. Figure 4(a) illustrates the initial charge-discharge curves of the samples between 2.8 V and 4.3 V at 0.5C rate. All cells had similar charge and discharge curves. When the pressure increase from 0 MPa to 6 MPa, the initial discharge capacities were measured as 172.6 mAh/g, 188.1 mAh/g, 201.2 mAh/g and 176.3 mAh/g, respectively. The discharge capacity of sample S4 was improved by 16.57% when compared to that of sample S0. The reason for such improvement can be ascribed to the reduced disordering of cation position within S4 lattice, as illustrated previously in Table 1 by XRD refinement. Furthermore, the relationships among the treat pressure, the diffraction intensity ratio, the cationic disordering and the specific capacity were summarized in Fig. 4(b), where a negative correlation was observed. It is clear that the pressure treatment can significantly improve the electrochemical performance of the cathode materials by decreasing the Li and Ni mixing.

Figure 4(c) shows the cyclic performance of all samples at 5C rate and 25 °C. After 100 cycles, the capacity for the four samples is 59.6 mAh/g, 102.4 mAh/g, 124 mAh/g and 103.8 mAh/g, the capacity retention is determined as 38.88%, 65.73%, 79.08% and 67.89%, respectively. Clearly, the S0 sample showed a large capacity fading after 100 cycles, whereas the S2, S4 and S6 samples exhibited enhanced cycling performance. Among the four samples, sample S4 presented the best cyclic performance at high rate condition. The reason may be ascribed to the reduction of cationic mixing, which increases the diffusion coefficient of lithium ions, and thus is beneficial for the improved cycle stability of the prepared materials. Figure 4(d) illustrates the rate capability of all samples. All the electrodes showed a decrease in capacity with the increased discharge rates. It also can be seen from the figure the capacity of pressing treated samples dropped more slowly, exhibiting improved capacity retention over the C rate range. Especially, at 5C rate, the capacity of S4 sample increased 9.66%, 3.25% and 3.92% compared with other three samples, respectively. We noticed some earlier works to improve the rate performance of NCA materials by surface coating, the AlF_3_^22^ coated NCA presented a capacity of 112 mAh/g at 5C rate, with capacity retention of 62%. In our case, the specific capacity for Sample S4 is 157 mAh/g, and the capacity retention is 78% respectively. It seems that our approach, the pressing treatment of the precursor powder, is also an effective way to reduce the cationic mixing and therefore improves the electrochemical performance.

**Figure 5:**
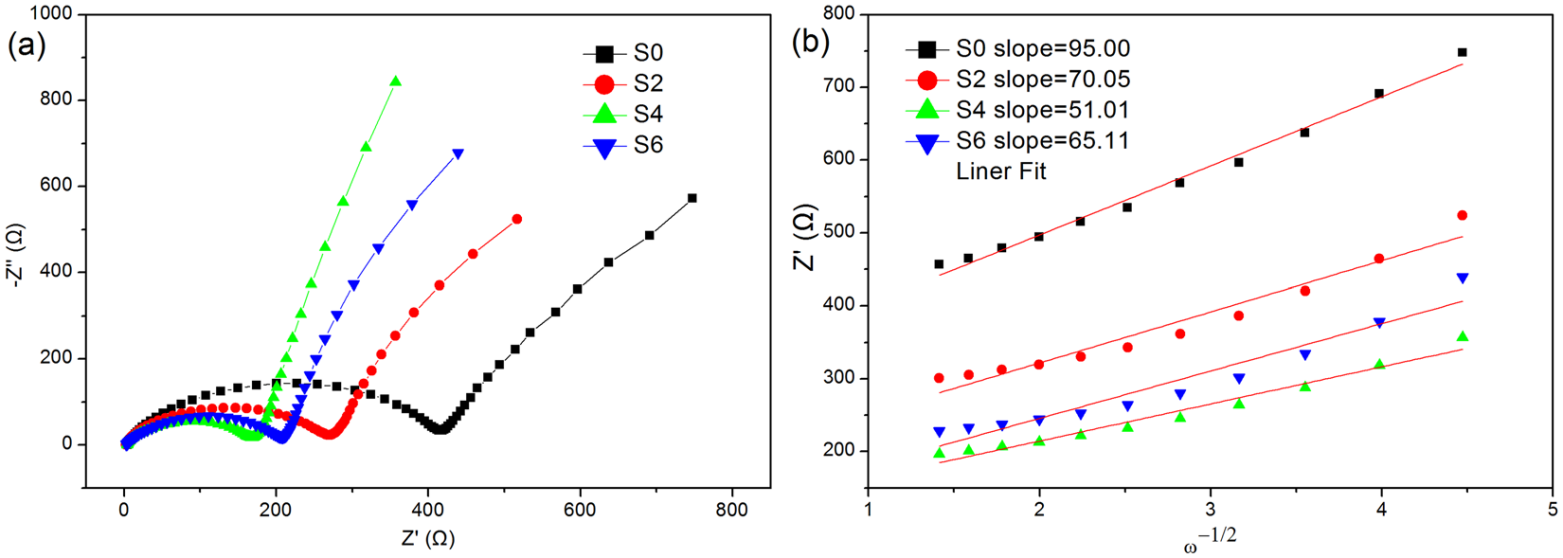
EIS results of electrodes. (**a**) Nyquist plot of the EIS of coin cells, (**b**) the linear relationship between the Warburg impedance and the inverse square root of angular frequency, the slopes of the simulated lines are the Warburg constant for the samples.

In order to further verify the reasons for the improvement of electrochemical performance, electrochemical impedance test were conducted for different samples. Figure 5(a) shows the Nyquist plots of all samples in 25 °C. There are semicircles at high frequencies and an oblique line at low frequencies. Such impedance spectra can be explained by an equivalent circuit model as shown in the inset of Fig. 5(a), in which a solution resistance (Rs), a charge-transfer resistance (Rct), a constant phase element (CPE) and Warburg impedance (Zw) were employed. The fitting process was carried out with Nova1.01 software, and the results are listed in Table 3. Among those parameters listed, Rct is most important since it represents the charge transfer within the cathode material. After going through the simulation, the Rct value of the four sample electrodes were 406 Ω, 262 Ω, 166 Ω and 260 Ω, respectively. The sample S4 showed the lowest charge transfer resistance, indicating that the charge transfer at the electrolyte/electrode interface is greatly enhanced after the pressing treatment.

**Table 3:** The values of Rs, and Rct for the Nyquist plots and calculated Li diffusion coefficient (D_Li_) for the samples of different pressure at 25 °C.

Sample	Rs(Ω)	Rct(Ω)	σ(Ω·s^−0.5^)	D_Li+_(cm^2^·s^−1^)
S0	3.86	406	95.00	1.26 × 10^−11^
S2	2.71	262	70.05	2.31 × 10^−11^
S4	3.41	166	55.15	4.36 × 10^−11^
S6	2.31	260	65.11	2.68 × 10^−11^

Li-ion diffusion coefficient can also be obtained with the data from impedance spectra^23^. The diffusion coefficient can be calculated according to the following equation (1):

1 $${\rm{D}}=\frac{{R}^{2}{T}^{2}}{2{A}^{2}{n}^{4}{F}^{4}{c}^{2}{\sigma }^{2}}$$

In the above equation, R is the gas constant, T is the absolute temperature, A is the effective working area of the cathode, n is the electronic transport ratio during redox process, F is the Faraday constant, c is taken as the molar density of Li-ion in an electrode, the σ can be obtained by the equation (2):

2 $${\rm{Z}}^{\prime} ={R}_{D}+{R}_{L}+\sigma {\omega }^{-1/2}$$

Figure 5(b) shows the linear relationship between the Warburg impedance and the inverse square root of angular frequency in low frequencies. The slopes of the simulated lines are the Warburg constant σ for the samples. Combining equation (1) and equation (2), the Li-ion diffusion coefficients can be calculated, and the results are list in Table 3.

The Li diffusion coefficient calculated value is generally in agreement with the reports from Duan, J *et al*.^3^ and Lee, MJ *et al*.^24^ Generally, a high D_Li_ value implies a well ordered layer structure of the cathode material. The D_Li_ of sample S4 in our case is 4.36 × 10^−11^ cm^2^·s^−1^, approximately 3.5 times that of sample S0, implying that the pressing treatment can effectively accelerate the migration of Li ions in the crystal. Therefore, the enhanced electrochemical performance of the cathode materials in our case can be regarded as the increased Li ion diffusion caused by the reduced cationic mixing in Li slab.

**Figure 6:**
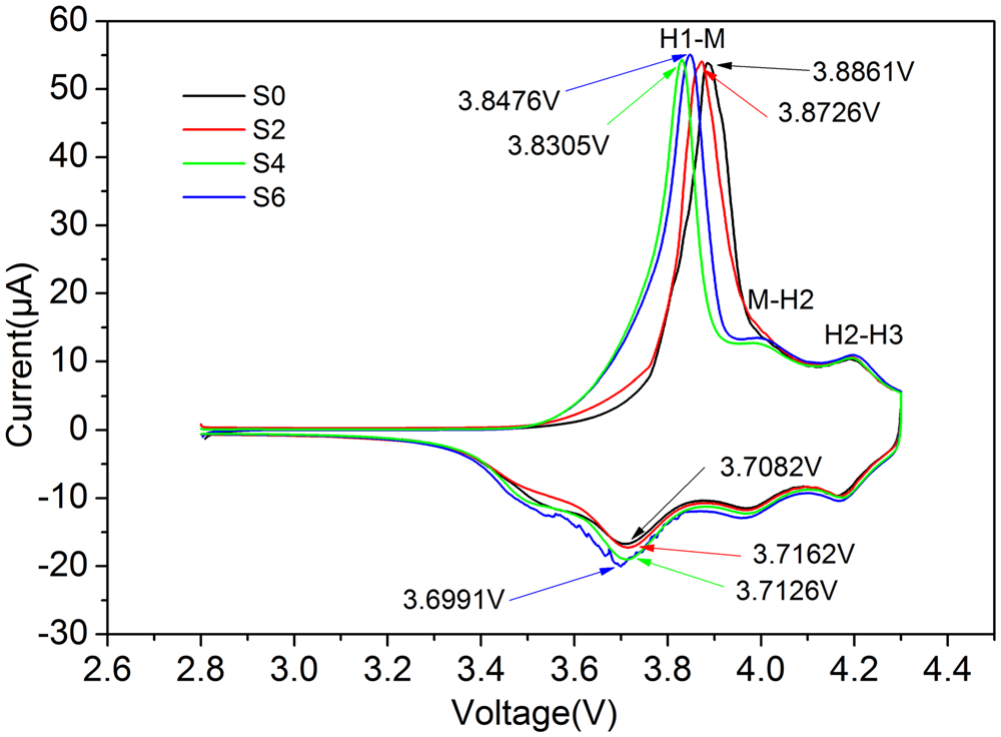
Cyclic voltammograms obtained with scan rate of 0.1 mv·s^−1^ for all samples.

Figure 6 shows the CV curves of samples between 2.8–4.3 V at a scan rate of 0.1 mVs^−1^ with fresh cell. As can be seen from the figure, all samples show three pairs of reduction and oxidation peaks, which are caused by the complex three phase transitions of hexagonal phase to monoclinic phase (H1-M), monoclinic phase to hexagonal phase (M-H2), and hexagonal phase to hexagonal phase (H2-H3) during the extraction and insertion of lithium ion. Such findings are consistent with the earlier reports from Xie, H^19, 25^ and Zhou, P^26^. In the H1-M redox peaks, the sample of redox voltage gap is 0.1779 V, 0.1564 V, 0.1179 and 0.1485 V, respectively. Such narrow voltage gap of S4 sample indicates that Sample S4 has a better reversibility, which consistent with the results of previous cyclic performance investigation.

## Conclusions

In summary, a simple synthesis routine was presented to prepared submicron sized NCA cathode materials with improved electrochemical performance. The powders were obtained by calcinating the precursor powders after a pressing treatment. XRD refinements showed that such pressing treatment is an effective way to increase the intensity ratio and reduce the cationic disordering in Li slab. The electrochemical testing showed improved electrochemical behaviors for those pressing treated samples. The initial discharge capacity of the sample S4 is 201.2 mAh/g, showing an increase of 16.57% when compared with the sample S0 without pressing treatment. S4 sample also showed excellent performance at high rate. At 5C rate, it delivered capacity retention of 79.08% after 100 cycles, while sample S0 only provided capacity retention of 38.88%. Such enhanced electrochemical performance can be ascribed to the increased Li ion diffusion caused by the reduced cationic disordering in Li slab. The S4 sample presented a diffusion coefficient of 4.36 × 10^−11^ cm^2^·s^−1^, which is almost 3.5 times the value of untreated sample. The submicron sized NCA materials obtained by the so called pressing treatment routine illustrated very attractive electrochemical performance for future application.
